# Determinants of stress coping behaviors in patients with Multiple Sclerosis (MS-DSCB): development and psychometrics of a PRECEDE model-based questionnaire

**DOI:** 10.1186/s12888-022-04217-2

**Published:** 2022-08-30

**Authors:** Zahra Hosseini, Atefeh Homayuni, Amin Ghanbarnejad

**Affiliations:** 1 Health Education and Health Promotion, Social Determinants in Health Promotion Research Center, Hormozgan Health Institute, Hormozgan University of Medical Sciences, Bandar Abbas, Iran; 2 Student Research Committee, Hormozgan University of Medical Sciences, Bandar Abbas, Iran; 3 Department of Public Health, School of Health, Social Determinants in Health Promotion Research Center, Hormozgan Health Institute, Hormozgan University of Medical Sciences, Bandar Abbas, Iran

**Keywords:** Stress, Factor Analysis, Psychometrics, PRECEDE Model, Multiple Sclerosis

## Abstract

**Objective:**

Stress management delays the onset or exacerbation of symptoms of multiple sclerosis. The present study aimed to develop and psychometrically evaluate a questionnaire to measure the determinants of stress coping behaviors in patients with multiple sclerosis.

**Methods:**

This was a methodological study that was conducted in two stages: qualitative and quantitative phases. Participants in this study were patients with multiple sclerosis who referred to the MS Association and Charity Foundations for Special Diseases in Isfahan in 2021. Preliminary item pool was developed by qualitative part of the study. The validity of the questionnaire was determined with item impact, content validity ratio (CVR), content validity index (CVI), face validity, exploratory factor analysis (EFA) and confirmatory factor analysis (CFA).

**Results:**

In the first stage, an item pool containing 97 items were generated and after removing duplicate items and merging some of them, a questionnaire containing 51 items was developed.

Ten items were removed based on the results of face validity and content validity. The EFA revealed 11 factors containing 41 items that explained 64% of the total variance of test. In CFA, 9 other items were deleted, and the questionnaire was reduced to 32 phrases in general. The results of the CFA determined the 9-factor structure of the questionnaire including awareness, attitude, self-efficacy, access to resources, skills of using resources, social support, important others, behavioral consequences and social comparison. The Cronbach's alpha coefficient of the questionnaire was 0.726.

**Conclusion:**

The results showed that the designed questionnaire is a valid and reliable tool for assessing the determinants of stress coping behaviors in patients with multiple sclerosis. Identifying these factors and designing interventions based on them, in order to control or reduce stress in these patients, can help to improve the quality of life in these patients.

**Supplementary Information:**

The online version contains supplementary material available at 10.1186/s12888-022-04217-2.

## Introduction

Nowadays, stress is considered as an exacerbating factor and a possible cause of multiple sclerosis (MS) [1]. Stress refers to any environmental, social, biological, or psychological demand that requires a person to adjust his or her usual patterns of behavior. Stress was conceptualized as exposure to noxious environmental stimuli such as extreme temperature [2].

Evidence supports the hypothesis that there is an association between stressful life events and an increased risk of exacerbating MS. Charcot suggested that stress may trigger disease activity [3]. Stress can exacerbate the immunity and inflammation of the brain, which is crucial in the pathogenesis of MS [4]. Patients with MS have reported that psychological stress can exacerbate their symptoms [5]. Kurt et al. [6] concluded that by controlling stressful life events and limiting their effects, the onset and exacerbation of MS can be delayed. Poser [7] also argues that controlling stress does not cure MS disease, but plays an important role in reducing its symptoms and severity. Given the importance of stress management and control in these patients, the use of coping behaviors in these patients is crucial. Identifying the factors affecting the performance of stress coping behaviors helps health promotion professionals to develop programs related to appropriate interventions to reduce and control stress in these patients.

In general, the issues of health education and the application of theories are raised when the need to change human behavior in the area of health is spoken [8]. Theories provide valuable tools for recognizing and solving a wide range of behavioral problems. There are several theories of health behavior in the scientific literature, each of which attempts to explain why people do a behavior successfully or fail to do that behavior [9]. The PRECEDE model is one of the most well-known and common planning models in health education and health promotion, which is used to analyze the determinants of behavioral factors. It includes five stages of social diagnosis, epidemiological diagnosis, behavioral diagnosis, educational diagnosis and evaluation [10]. In the educational diagnosis stage of this model, predisposing, enabling and reinforcing factors that are effective in adopting stress coping behaviors are identified [11].

A review of the literature on the tools designed in this area revealed that a scientific standard questionnaire has not yet been designed to assess the determinants of stress coping behaviors in patients with MS. Thus, the present study was conducted with the aim of 1) designing an appropriate tool based on the PRECEDE model for identifying the factors affecting the adoption of the stress coping behaviors in this group of patients, 2) determining the psychometrics of this tool.

## Materials and methods

### Research design and population

The present study is a methodological research that was conducted in both qualitative (design and development) and the quantitative stages (the assessment of psychometric properties of the instrument being developed). Methodological research includes defining the concept or behavior being measured, forming tool items, and finally examining the validity and reliability of the tool [12].

This study was conducted from April 2020 to March 2021 and its statistical population was all patients with MS who referred to the MS Association and Charity Foundations for Special Diseases in Isfahan.

### Section 1: design and item generation

As a first step in this process, semi-structured interviews were conducted in the form of a qualitative directed content analysis approach with 26 patients with MS who referred to the MS Association of Isfahan. The inclusion criteria of individuals with MS were: 1) having MS diagnosed by a neurologist, 2) having MS for more than 1 year, 3) having not a chronic disease other than MS, and 4) being able to participate in the interview and sharing their experiences. Individuals were excluded if they were unable to cooperate and talk due to the worsening of the disease or other reasons and were not willing to continue the interview at the time of the interview. We have tried to recruit patients with different characteristics to ensure that diverse demographic backgrounds are present in the interviews.

The mean age of the participants was 36.11 (25–49 years). In relation to their marital status, 53.85% were married, while 26.92% were single. In connection with respondents' level of education, the majority of participants had high school and diploma degree as their highest qualifications (50%). The results further indicated that most of the participants (84.61%) were housewife. All patients received health insurance services.

In this process, given the aim of the study, data were collected to identify behavioral and non-behavioral factors affecting the adopting stress coping behaviors in patients with MS based on the PRECEDE model [13]. The interview consisted of the following open-ended questions: 1) What do you know about stress? 2) Do you know about the consequences of stress? 3) Tell me about your skills and abilities to cope with stress. 4) In your opinion, what skills should you learn to control stress? 5) Tell me about your experiences regarding environmental barriers to cope with stress. 6) Tell me about your experiences regarding the role of family members, health care providers, friends, and others in performing stress coping behaviors. 7) Tell me about your feelings after doing stress coping behaviors continuously. 8) What problems did you have, when performing stress coping behaviors?

Each interview lasted for 30–90 min. The interviews were held in the patient's living place or in a dedicated room in the MS Association. We stopped data collection until the saturation was reached. Interviews were entered into MAXQDA software and data were analyzed and categorized.

The basic draft of the questionnaire was designed based on the data gathered through individual interviews, which included 97 items. These items were reviewed by the research team in several sessions to ensure non-overlap as well as non-duplication and were finally approved. Some duplicate items were removed and items that could be merged were merged with each other and some items were modified. Finally, after the final review, the initial proposed tool was approved by the members of the research team with 51 items.

### Section 2: tool psychometrics

This section was implemented in three steps as follows:

#### Step 1: examining the face validity and content validity

##### Face validity

Face validity means whether the test participants agree with the items and wording of the tool to achieve the research objectives [14]. In this study, quantitative and qualitative methods were used to determine face validity. In qualitative stage, face-to-face interviews were conducted with 10 patients to assess each item for ambiguity, relevancy and difficulty. In the next step, to reduce and eliminate inappropriate items and determine the importance of each item, the quantitative method of item impact was used [15]. In this step, for each item of questionnaire, a 5-point Likert scale was considered, including very important (score 5), somewhat important (score 4), relatively important (score 3), slightly important (score 2), does not important at all (score 1). Then, 10 patients were asked to examine each item and determine the importance of each item based on the 5-point Likert scale. Then, the score of each item was calculated separately based on the following formula:

$$Impact\mathit\;Score\mathit=\mathit\;Frequency\mathit\;\mathit(\mathit\%\mathit)\mathit\;\mathit\ast\mathit\;importance$$

If the impact score was 1.5 or above, the item was identified and maintained for further analysis.

##### Content validity

In the content validity, to ensure that the test represents the construct that is claimed to measure, the content of the test is evaluated [16]. Content validity was assessed by both quantitative and qualitative methods. In the qualitative phase, a panel of 10 experts in health education and health promotion, psychology and counseling, neurologists and experts in the area of instrumentation were asked to evaluate the questionnaire for grammar, wording, item allocation and scaling indices. They checked all items and inserted their recommendations into the questionnaire. Content validity ratio (CVR) and content validity index (CVI) were used to confirm the quantitative content validity. To calculate the CVR index, experts were asked to assess the necessity of each item using a 3-point rating scale (item is necessary, item is useful but not necessary, and item is not necessary). CVR was calculated through the following formula:

$$CVR=\frac{\;{}^nE-{\displaystyle\frac N2}}{\displaystyle\frac N2}$$

In this formula, n_E_ is the number of experts chosen optionally (it is necessary) and N is the number of whole experts. Based on the Lawshe's table and the number of the experts (*n *= 10), the value of 0.62 was considered as the minimum acceptable value for content validity ratio.

To examine the content validity index (CVI), three criteria of simplicity, relevance and clarity were assesses separately and in a 5-point Likert scale for each item by the relevant experts [17]. CVI was calculated through the following formula:

$$\mathrm{CVI}\mathit=\mathit\;\frac{Number\mathit\;of\mathit\;raters\mathit\;chosing\mathit\;points\mathit\;\mathit3\mathit\;and\mathit\;\mathit4}{Total\mathit\;number\mathit\;of\mathit\;raters}$$

A score of 0.79 and higher for each item led to the acceptance of the item.

#### Step 2: Examining the construct validity

The construct validity of the questionnaire was performed using both exploratory (EFA) and confirmatory factor analysis (CFA). Exploratory factor analysis was applied to determine the underlying constructs of the questionnaire. Factor loadings equal or greater than 0.3 were considered appropriate. Confirmatory factor analysis was performed for comparing and assessing the model fitness [18, 19]. Several indicators must be considered to identify a model's fitness including: relative Chi-square, Root Mean Square Error of Approximation (RMSEA), Comparative Fit Index (CFI), Incremental Fit Index (IFI), Tucker-Lewis Index (TLI) [19–21].

The Kaiser–Meyer–Olkin (KMO) test was utilized for sample size adequacy, and Bartlett's Test of Sphericity was used to assess the appropriateness of the data. The recommended value of KMO for doing factor analysis on data is between zero and 1.

### Sampling method

In this study the size of the population (including patients with MS who refereed to the MS Association and Charity Foundations for Special Diseases in Isfahan, which have the inclusion criteria) is about 2000 people. According to the initial study with the size of 51 people in this research, the standard deviation was 0.305 and d = 0.04 and using the below formula, a sample size of 201 anticipated for the study.

$$n=\;\frac{N\;\times\;\sigma^2\;\times\;Z_{1-{\displaystyle\frac a2}}^2\;}{\left(N-1\right)\;\times\;d^2\;+\;\sigma^2\;\times\;Z_{1-{\displaystyle\frac a2}}^2}$$

Regarding to the existing limitations due to the outbreak of Covid-19 and the impossibility of distributing questionnaires in paper form and for protecting the health of participants, online questionnaires were designed to collect the data. The questionnaire was designed virtually and the link of the questionnaire was placed on the Telegram channel of the MS Association and Charitable Associations. Necessary and brief explanations about the objectives of the research were provided to the participants and they were asked to assist the researchers in conducting the research by completing this questionnaire. Participants had to answer all questions and after that they registered their answers by clicking the submit button. As a result, there was not missing data. To emphasize on the greater participation of individuals in the study, messages and links to participate in the study were resent as a reminder two weeks after the first submission.

In the present study, the inclusion criteria were: 1) having MS diagnosed by a neurologist, 2) having MS for more than 1 year, 3) having not a chronic disease other than multiple sclerosis, 4) willingness to participate in the study and 5) internet access to answer the questions. Patients were excluded if they lost any of the inclusion criteria.

Demographic information of the participants in this stage is summarized in Table 1. The mean age of the participants was 36.03 years.

**Table 1:** Demographic characteristics of participants

Variable	N (Percentage)
Gender	
Male	24(11.65)
Female	182(88.35)
Marital status	
Single	80(38.83)
Married	108(52.43)
Widow	5(2.43)
Divorced	13(6.31)
Education level	
Junior high school	9(4.37)
High school and diploma	63(30.58)
Associate degree	19(9.22)
Bachelor’s degree and higher	115(55.83)
Occupation status	
Housewife	127(61.65)
Employed	52(25.24)
Student	15(7.28)
Disabled	7(3.4)
Retired	5(2.43)

#### Step 3: examining the reliability

Reliability of a tool indicates the accuracy of its measurement. Reliability refers to the internal consistency and stability in measuring the attributes or constructs of a tool [22]. In the present study, internal consistency method was used to determine the reliability of the questionnaire. In this method, the tool is presented to the participants and then the correlation between the questions is calculate using Cronbach's alpha.

Data were analyzed using SPSS-24 and AMOSS-22 software.

### Ethical considerations

The present research was approved by the Ethics Committee of Hormozgan University of medical sciences (IR.HUMS.REC.1399.065). Participants completed the questionnaire voluntarily and were assured that their information would remain confidential and they could withdraw from the study at any time.

## Results

### Content and face validity

After calculating the item impact score index, since the values ​​of this index for tool items were higher than 1.5, none of them were removed and all of them were considered important and appropriate for the target group and were maintained for the next steps. The results of calculating the content validity ratio showed that the values ​​of the content validity ratio for 10 items were lower than the presented value in the Lawshe's table (0.62) and these items were removed from list of items. Therefore, 41 items remained to conduct the next step.

### Construct validity

In this research, a questionnaire with 41 questions was used and the Cronbach's alpha coefficient showed a desirable internal consistency. In the following, the results of exploratory factor analysis are presented.

The Kaiser–Meyer–Olkin and Bartlett's test of Sphericity results showed the adequacy of samples for factor analysis and it indicates the existence of correlation between the variables and the appropriateness of the data for factor analysis (Table 2).

**Table 2:** KMO and Bartlett’s Test

Kaiser–Meyer–Olkin Measure of Sampling Adequacy		.707
Bartlett's Test of Sphericity	Approx. Chi-Square	3359.345
	df	820
	Sig	.000

In the current exploratory factor analysis, 41 questions in 11 factors have an eigenvalue above 1, so 11 factors can be obtained, which accounted for 64% of observed variance. Now we need to know what questions does the created 11 factors include? Therefore, the principel component analysis with varimax rotation is used. The classification of factors is presented in Table 3.

**Table 3:** Classification of questions

Factor	Question
Attitude	4–7-8–13-14–15-16–17
Behavioral consequences	27–28-29–30
Social comparison	37–38-40–41
Self-efficacy	5–6-9–10-11–12
Skills of using resources	24–25-26
Awareness	1–2-3
Important others	34–35-36
Existence of resources	18–19-20–21
Social support	31–32-33
Access to resources	22–23
thanksgiving	39

Table 4 showed the reliability of each factor. Regarding the factor number 4 (self-efficacy) it is necessary to delete questions 6 and 9.

**Table 4:** Reliability of the dimensions of questionnaire

Factor	Cronbach’s alpha coefficient	Number of questions
Attitude	0.815	8
Behavioral consequences	0.873	4
Social comparison	0.811	4
Self-efficacy	0.736	4
Skills of using resources	0.79	3
Awareness	0.785	3
Important others	0.667	3
Existence of resources	0.627	4
Social support	0.629	3
Access to resources	0.705	2
Thanksgiving		1

**Fig. 1:**
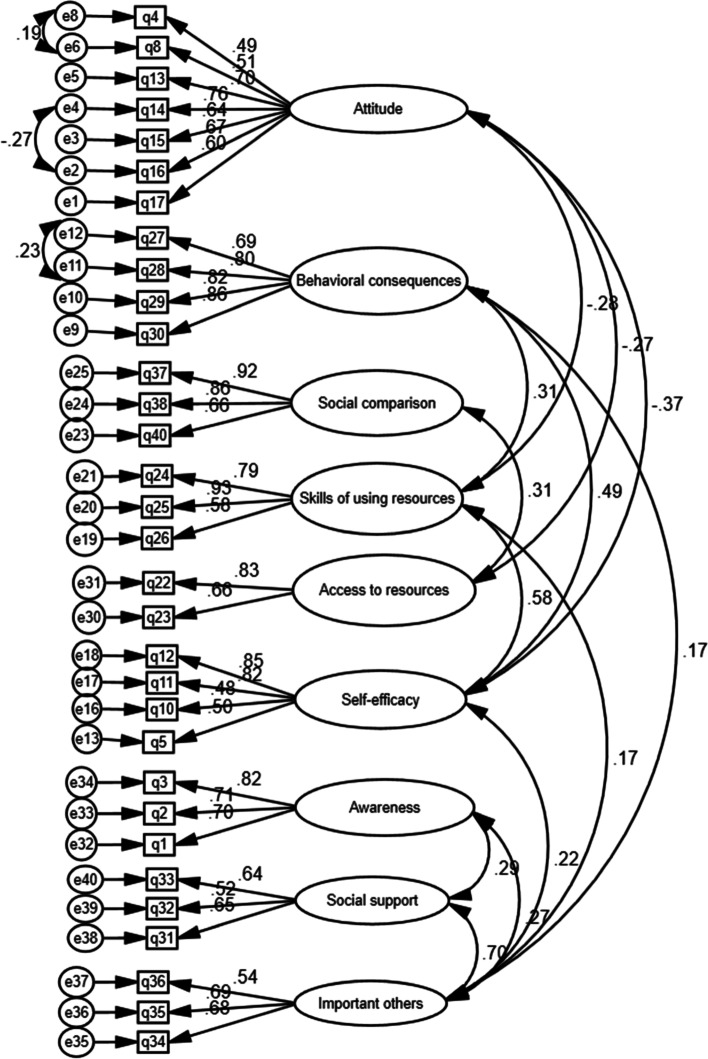
The results obtained from confirmatory factor analysis

In this section, the results of confirmatory factor analysis are presented. Figure 1 shows the structural equations of the confirmatory factor analysis.

Table 5 showed the goodness-of-fit tests of the general model. These findings showed that the model fits the data well. The chi-square test showed the fit of the model with the variance–covariance matrix. Other suitability criteria were also checked, such as RMSEA = 0.045 that was not suitable. The statistics of TLI, IFI, and CFI were more than 0.9, and in general, all the fit criteria of the overall model confirmed the appropriateness of the fit.

**Table 5:** Fit indices of confirmatory factor analysis for model

	TLI	IFI	CFI	RMSEA	CMIN	DF	P	CMIN/DF
The general model	0.912	0.922	0.920	0.045	635.000	448	0.000	1.417

Covariance and variance between factors that are significant were presented in Table 6, and non-significant covariances have been removed.

**Table 6:** Covariance and correlation

			Estimate	S.E	C.R	*P*	Correlation
ATTITUDE	< – >	SKILLS OF USING RESOURCES	-.105	.032	-3.231	.001	-.283
ATTITUDE	< – >	ACCESS TO RESOURCES	-.122	.044	-2.763	.006	-.267
ATTITUDE	< – >	SELF-EFFICACY	-.202	.047	-4.334	***	-.373
BEHAVIORAL CONSEQUENCES	< – >	SELF-EFFICACY	.318	.057	5.590	***	.494
BEHAVIORAL CONSEQUENCES	< – >	IMPORTANT OTHERS	.033	.016	2.093	.036	.173
SOCIAL COMPARISON	< – >	ACCESS TO RESOURCES	.304	.099	3.063	.002	.311
SELF-EFFICACY	< – >	SKILLS OF USING RESOURCES	.332	.063	5.235	***	.577
SELF-EFFICACY	< – >	IMPORTANT OTHERS	.054	.020	2.646	.008	.218
AWARENESS	< – >	ATTITUDE0	.077	.028	2.723	.006	.287
AWARENESS	< – >	IMPORTANT OTHERS	.065	.024	2.669	.008	.272
IMPORTANT OTHERS	< – >	ATTITUDE0	.059	.013	4.485	***	.703
BEHAVIORAL CONSEQUENCES	< – >	SKILLS OF USING RESOURCES	.135	.038	3.532	***	.306
SKILLS OF USING RESOURCES	< – >	IMPORTANT OTHERS	.028	.014	2.057	.040	.167
e11	< – >	e12	.080	.034	2.388	.017	.233
e2	< – >	e4	-.100	.037	-2.744	.006	-.266
e6	< – >	e8	.099	.039	2.509	.012	.192

The final version of the questionnaire consisted of 32 items in 9 dimensions: awareness (3 items), attitude (7 items), self-efficacy (4 items), access to resources (2 items), skills of using resources (3 items), social support (3 items), important others (3 items), behavioral consequences (4 items) and social comparison (3 items). The awareness construct measured participants' awareness about the sources of stress, its symptoms and stress coping strategies. Participants could choose any number of options that they thought were correct. The minimum score was 3 and the maximum was 15. Higher score indicated higher participants' awareness. The attitude construct was developed to assess the positive and negative attitudes of participants to the stress, and measured based on a 5-point Likert scale ranging from strongly agree to strongly disagree. The minimum score was 7 and the maximum was 35. Higher score indicated more negative attitudes of participants toward the stress. The self-efficacy construct assessed the beliefs of the participants about their ability to cope with stress. The minimum score was 4 and the maximum was 20. Higher score indicated higher self-efficacy of participants to cope with stress. The enabling factors consisted of 2 constructs, assessed participants' skills and accessibility of resources to do stress coping behaviors. The questions of this section were designed in a 5-point Likert scale ranging from strongly agree to strongly disagree. The minimum score was 5 and the maximum was 25. The reinforcing factors consisted of 3 constructs including social support, important others and behavioral consequences. In behavioral consequences construct, the participants were asked about the behavioral consequences of performing stress coping behaviors. The questions of this section were designed in a 5-point Likert scale ranging from strongly agree to strongly disagree. The minimum score was 4 and the maximum was 20. In two other constructs, participants were asked about the extent of family, health care professionals and friends' supports to do coping behaviors. The minimum score was 0 and the maximum was 6. And finally, social comparison assessed how the participant compares himself/herself with other patients. The questions were to be rated in a 5-point Likert scale ranging from strongly agree to strongly disagree. The minimum score was 3 and the maximum was 15.

## Discussion

The aim of the present study was to design a questionnaire for measuring the factors affecting the adoption of the stress coping behaviors in patients with MS. This questionnaire was developed using a qualitative study and continued by removing items, testing them and reviewing the questionnaire. The results of the present study approved the reliability and validity of the designed questionnaire. According to the results of the study, 32 items were approved after examining face validity, content validity, and construct validity. Then, they were divided into 4 groups of predisposing factors, enabling factors, reinforcing factors and social comparison.

Predisposing factors indicate that adopting or changing a behavior requires a number of factors that precede the change of behavior and provide motivation to perform a behavior and cause the logic of that behavior. These factors in our study included three factors: awareness, attitude and self-efficacy. Enabling factors indicate that a number of preconditions are required to adopt or change a behavior. These preconditions are considered a behavioral or environmental change that creates motivation before doing that behavior and affects one’s behavior directly or indirectly through environmental factors. In the present study, these factors were obtained in the form of two subcategories as: access to resources and skills of using resources. Reinforcing factors state that for a behavior to continue, be repeated, and reinforced, it needs a number of factors to continuously provide a reward for maintaining the behavior and ultimately lead to the stability and continuity of that behavior. In this study, these factors were obtained in the form of three subcategories: social support, important others and behavioral consequences. The novel contribution of the current study relies on the fact that we added a social comparison on the PRECEDE model. Comparison of self with others, referred to as social comparison, is among the factors that can increase or decrease participants’ motivation to perform stress coping behaviors [13]. This finding is consistent with the results of studies conducted by Alizadeh-Siuki et al. [23], Ghasemi et al. [24] Nazari et al. [25] and Nahidi et al. [26].

Alizadeh-Siuki et al. [23] examined the psychometric properties of a questionnaire on brucellosis prevention behaviors based on the PRECEDE model among rural farmers and their family members. In their study a questionnaire with 36 items and 8 subscales including knowledge, attitude, self-efficacy, social support, enabling factors, environmental factors, behavioral factors and reinforcing factors developed. The designed questionnaire's exploratory factor analysis in study conducted by Ghasemi et al. [24] revealed four factors: self-efficacy, attitude, reinforcing factors and enabling factors. These four factors explained 57.51% of the total variance of the test. The final developed questionnaire in study conducted by Nazari et al. [25] included 25 items in three dimensions: knowledge, attitude and enabling factors. This questionnaire was developed to evaluate the behavioral factors affecting musculoskeletal disorders among adolescent students. In addition, the designed questionnaire's' exploratory factor analysis by Nahidi et al. [26] lead to identifying 15 factors and 3 constructs including predisposing factors, enabling factors and reinforcing factors. This questionnaire was developed for measuring factors associated with mother-newborn skin-o-skin contact based on the PRECEDE-PROCEDE model.

According to the results, the designed questionnaire has good validity and reliability to measure the factors affecting the adoption of stress coping behaviors in the target group. According to the literature review, it seems that no tool has been designed to measure the these factors, although some questionnaires have been designed to examine the factors related to different behaviors using the PRECEDE model [23–28].

CVI and CVR indices were used to assess the content validity of the questionnaire. The obtained results indicate the validity of the questionnaire in terms of these two mentioned indices. After performing content confirmatory analysis, 10 items were removed. Evaluation of Cronbach's alpha coefficient of the questionnaire indicate the acceptability of this tool. This result indicates that this questionnaire can provide reliable results in different temporal and spatial conditions [29] and each construct measures the same subject [30].

Extracting items from patients' point of view and qualitative research and conducting a mixed-method study to design a questionnaire were among the strengths of this study. One of the limitations of the study was sampling from one city and completing questionnaires in a self-reporting manner. Also, since participants were invited to participate through a convenience sampling method, the generalizability of the results may be limited.

## Conclusion

The present study results led to development of a standard and comprehensive questionnaire to assess the factors affecting the adoption of the stress coping behaviors in patients with MS based on the PRECEDE model. The proposed questionnaire had good psychometric properties and could be used as a valid and practical tool to assess the factors related to performing stress coping behaviors. Researchers can use this tool to evaluate the effectiveness of intervention programs to reduce and control stress in these patients.

## Supplementary Information

**Additional file 1: **The initial version of MS-DSCB.

## Data Availability

The datasets used analyzed during the current study are not publicly available due the possibility that sharing interviews, which contain sensitive information about participants' identities, may compromise participant anonymity, however, the quantitative data are available from the corresponding author on reasonable request.
