# Supplementary material for: Determinants of stress coping behaviors in patients with Multiple Sclerosis (MS-DSCB): development and psychometrics of a PRECEDE model-based questionnaire
Source: BMC Psychiatry. 2022 Aug 30;22:578. doi: 10.1186/s12888-022-04217-2 (PMC9426368; doi:10.1186/s12888-022-04217-2)
Supplement: Supplementary file 1 — Additional file 1: The initial version of MS-DSCB. [file 12888_2022_4217_MOESM1_ESM.docx]

**Appendix 1: Questionnaire**

1)Which of the following can cause stress? (You can choose more than one option).

1. Not having enough time to do things
2. Physical diseases such as multiple sclerosis
3. Life changes such as marriages, death of a family member and so on.
4. Fear of the future, such as worrying about marriage, having children and so on.
5. Excessive punctuality

2) Which of the following items can be a sign of stress? (You can choose more than one option).

1. Increase of heart rate
2. Sweat
3. Breathing disorder (rapid breathing or shortness of breath)
4. Disquiet and distress

3) Which of the following methods can be used to reduce and cope with stress? (You can choose more than one option).

a) Leaving or avoiding stressful situations

b) Searching and receiving spiritual support (such as praying, trust in God, going to religious places and so on.)

c) Saying the affirmative sentences

d) Expressing feelings (talking about stressful experiences or writing them)

e) Voluntarily helping others or getting help from others

f) Doing the favorite hobbies (such as exercise and recreation, listening to pleasant music and so on).

4) I believe that stress causes my disease will recur.

a) Completely agree b) Agree c) No idea d) Disagree e) Completely disagree

5) I believe that stress is manageable.

a) Completely agree b) Agree c) No idea d) Disagree e) Completely disagree

6) I believe that some stresses can only be resolved over time.^*^

a) Completely agree b) Agree c) No idea d) Disagree e) Completely disagree

7) I believe that my family plays a big role in creating stress for me.^*^

a) Completely agree b) Agree c) No idea d) Disagree e) Completely disagree

8) I believe that stress disrupts my life.

a) Completely agree b) Agree c) No idea d) Disagree e) Completely disagree

9) I believe that coping with stress does not require special skills.^*^

a) Completely agree b) Agree c) No idea d) Disagree e) Completely disagree

10) I have the ability not to transfer my stress to others

a) Completely agree b) Agree c) No idea d) Disagree e) Completely disagree

11) I can cope with my stress and control it.

a) Completely agree b) Agree c) No idea d) Disagree e) Completely disagree

12) I can distract myself when I'm stressed.

a) Completely agree b) Agree c) No idea d) Disagree e) Completely disagree

13) If I am exposed to constant and severe stress, I will suffer from migraines and nervous headaches.

a) Completely agree b) Agree c) No idea d) Disagree e) Completely disagree

14) If I am exposed to constant and severe stress, I will be sad, angry or depressed.

a) Completely agree b) Agree c) No idea d) Disagree e) Completely disagree

15) If I am exposed to constant and severe stress, my ability to concentrate and pay attention will decrease.

a) Completely agree b) Agree c) No idea d) Disagree e) Completely disagree

16) If I am exposed to constant and severe stress, my sleep will be disrupted.

a) Completely agree b) Agree c) No idea d) Disagree e) Completely disagree

17) If I am exposed to constant and severe stress, I will overeat or, conversely, my appetite will decrease.

a) Completely agree b) Agree c) No idea d) Disagree e) Completely disagree

18) So far, the MS Association (Charity Foundations) has not held relaxation classes (yoga, meditation, etc.) for patients.^*^

a) Completely agree b) Agree c) No idea d) Disagree e) Completely disagree

19) The MS Association (Charity Foundations) organizes recreational camps with the aim of creating communication and empathy among patients.^*^

a) Completely agree b) Agree c) No idea d) Disagree e) Completely disagree

20) The MS Association provides me with the necessary information and training resources (such as book, quarterly and so on).^*^

a) Completely agree b) Agree c) No idea d) Disagree e) Completely disagree

21) My physician provides me with the necessary information and training resources (such as book, quarterly and so on).^*^

a) Completely agree b) Agree c) No idea d) Disagree e) Completely disagree

22) Due to traffic problems, it is difficult for me to participate in MS Association (Charity Foundations) counseling and sports programs.

a) Completely agree b) Agree c) No idea d) Disagree e) Completely disagree

23) Due to lack of time, it is difficult for me to participate in MS Association (Charity Foundations) counseling and sports programs.

a) Completely agree b) Agree c) No idea d) Disagree e) Completely disagree

24) I have "planning and time management" skills to cope with stress.

a) Completely agree b) Agree c) No idea d) Disagree e) Completely disagree

25) I have the "problem-solving" skill to cope with stress.

a) Completely agree b) Agree c) No idea d) Disagree e) Completely disagree

26) I have relaxation skills (such as deep breathing, meditation and yoga) to cope with stress.

a) Completely agree b) Agree c) No idea d) Disagree e) Completely disagree

27) Doing stress coping behaviors has made me sleep easier at night.

a) Completely agree b) Agree c) No idea d) Disagree e) Completely disagree

28) Doing stress coping behaviors gives me a feeling of calm and patience.

a) Completely agree b) Agree c) No idea d) Disagree e) Completely disagree

29) Doing stress coping behaviors reduces my stress and controls it.

a) Completely agree b) Agree c) No idea d) Disagree e) Completely disagree

30) I feel happy and cheerful by doing stress coping behaviors.

a) Completely agree b) Agree c) No idea d) Disagree e) Completely disagree

31) Are you invited to relaxation by others when you have stress?

a) Yes b) No

32) Do others help you in doing things when you are under stress due to lack of time?

a) Yes b) No

33) Are you encouraged by others to learn about stress and the ways of coping with it?

a) Yes b) No

34) Are you encouraged by others to do yoga and have entertainment?

a) Yes b) No

35) Are you encouraged by others to use relaxation techniques and deep breathing?

a) Yes b) No

36) Are you encouraged by others to express your feelings (talking about or writing about your stressful experiences) during times of stress?

a) Yes b) No

37) I avoid going to the MS Association because I know I get worse off when I see other patients.

a) Completely agree b) Agree c) No idea d) Disagree e) Completely disagree

38) I avoid going to the MS Association because I do not want to keep distance myself from a healthy society.

a) Completely agree b) Agree c) No idea d) Disagree e) Completely disagree

39) When I see that others are worse off than me, I thank God for my better condition.^*^

a) Completely agree b) Agree c) No idea d) Disagree e) Completely disagree

40) My spirit droop as I see that others are worse off than me.

a) Completely agree b) Agree c) No idea d) Disagree e) Completely disagree

41) When I am with my friends who have MS, I feel comfort because I think I am not alone.^*^

a) Completely agree b) Agree c) No idea d) Disagree e) Completely disagree

**(^*^= These questions were deleted in confirmatory factor analysis).**
